# Syncytiotrophoblast Functions and Fetal Growth Restriction during Placental Malaria: Updates and Implication for Future Interventions

**DOI:** 10.1155/2015/451735

**Published:** 2015-10-26

**Authors:** Winifrida B. Kidima

**Affiliations:** Department of Zoology, College of Natural and Applied Science, University of Dar es Salaam, P.O. Box 35064, Dar es Salaam, Tanzania

## Abstract

Syncytiotrophoblast lines the intervillous space of the placenta and plays important roles in fetus growth throughout gestation. However, perturbations at the maternal-fetal interface during placental malaria may possibly alter the physiological functions of syncytiotrophoblast and therefore growth and development of the embryo *in utero*. An understanding of the influence of placental malaria on syncytiotrophoblast function is paramount in developing novel interventions for the control of placental pathology associated with placental malaria. In this review, we discuss how malaria changes syncytiotrophoblast function as evidenced from human, animal, and *in vitro* studies and, further, how dysregulation of syncytiotrophoblast function may impact fetal growth *in utero*. We also formulate a hypothesis, stemming from epidemiological observations, that nutrition may override pathogenesis of placental malaria-associated-fetal growth restriction. We therefore recommend studies on nutrition-based-interventional approaches for high placental malaria-risk women in endemic areas. More investigations on the role of nutrition on placental malaria pathogenesis are needed.

## 1. Introduction

*Plasmodium falciparum* is known to cause the most severe form of malaria, a disease that claims the lives of about one million people annually [1]. The disease is severe in children under the age of five and in pregnant women. In pregnant women, malaria is associated with an increased risk of poor pregnancy outcomes, including maternal anemia, preterm delivery (PTD) (i.e., delivery before 37 weeks of gestation), and fetal growth restriction (FGR) which is defined as inability of the fetus to reach genetically predetermined size and weight at term [2]. Anemia, PTD, and FGR are major causes of malaria-associated low birth weight (LBW) babies. The prevalence of LBW babies as a result of placental malaria has been well documented in malaria endemic areas including Thailand, Papua New Guinea, and Sub-Saharan African countries. The overall prevalence of malaria-associated LBW babies from studies in endemic areas from year 1985 to 2000 has been estimated to be 20% of live births (reviewed in [3]). Studies show that infants born with LBW not only have increased risks of dying in the first year of life but have potential health problems in adulthood [4, 5]. Efforts to understand the mechanism of disease pathology that leads to poor pregnant outcomes are important for identifying targets for future intervention(s) and designing approaches that could lead to disease prevention.

**Figure 1:**
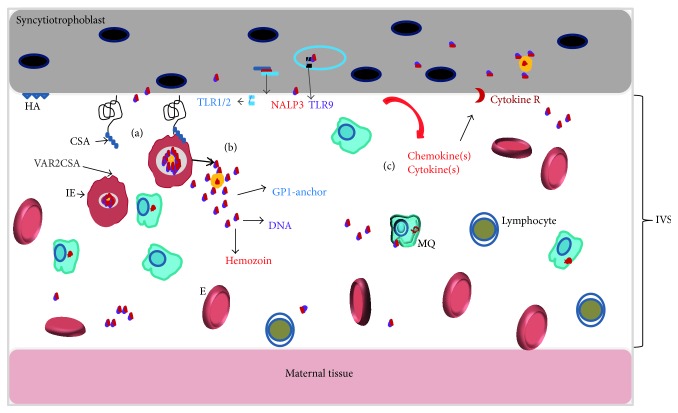
The microenvironment in the intervillous space of the placenta during active placental malaria: (a) Interaction of parasite ligand, VAR2CSA [10, 12] with CSA that is expressed by ST [8, 13]. (b) Recognition of parasite bioactive molecules of schizogony by surface PRRs expressed by both maternal macrophages and fetal syncytiotrophoblast; that is, malarial GPI-anchor bind TLR1/TLR2 or TLR2/TLR6 [14], and parasite's DNA is recognized by TLR9 [15] and hemozoin by TLR9 (see [16] and inflammasome (NALP3) [17]). (c) Inflammation in the IVS is attributed to chemokines and cytokines secreted by maternal macrophages, monocytes, and T cell as well as ST [15, 16, 18–20]. (HA: hyaluronic acid; CSA: chondroitin sulphate A; IVS: intervillous space; NALP3: inflammasome; GPI: glycosylphosphatidylinositol; IE: infected erythrocyte; E: erythrocyte).

Placental malaria changes the environment in the intervillous space of placenta (Figure 1). It occurs as a result of* P. falciparum* infected erythrocytes (IE) binding to syncytiotrophoblast (ST), a continuous, multinucleated, specialized epithelia layer that covers interior of the villous of the placenta.* P. falciparum*-IEs specifically bind on ST receptors known as chondroitin sulphate A (CSA) and hyaluronic acid [6–9]. Binding of IE to ST leads to sequestration of IE in the intervillous space (IVS) of the placenta. The ability of IE to bind on CSA is conferred by the parasite's variant surface antigen belonging to var. gene subfamily (VAR2) that is expressed on the surface of IE called VAR2CSA [10–12]. Generally, sequestration of IE in the IVS leads to secretion of chemokines resulting in inflammatory cells recruitment and cytokine production which is associated with poor pregnancy outcomes [18, 21, 22]. Although placental malaria is largely asymptomatic, there is a correlation between peripheral and placental parasitemia at delivery. Placental malaria can be categorized as acute, chronic, or previous infection using specific pathological criteria [23]. While chronic placental malaria has been associated with an increased risk of FGR, acute placental malaria has been associated with PTD in malaria endemic areas [24]. However, the major questions to date are the mechanisms on how chronic placental malaria induces FGR and PTD. In this review we discuss selected functions of ST and then highlight the role of malaria in dysregulation of these functions using evidence from animal, human, and* in vitro* studies. We then develop a model that describes the relationship between placental malaria FGR and the dysregulated syncytiotrophoblast function. We suggest a potential interventional approach targeting ST using evidence from epidemiological studies.

## 2. The Placenta and Its Response to Malaria

### 2.1. Maternal Responses to Malaria and Effect on the Placenta

Although pathogenesis of placental malaria is not completely understood, the IE binding to ST and recognition of parasite by maternal macrophages induce secretion of chemokines that recruit maternal monocytes into the IVS, resulting in inflammation. Studies show that there is high level of monocytes and macrophages in the IVS of the placenta during active placental malaria [25–28]. Earlier studies by Fried et al. [18] reported elevated levels of T-helper-1 cytokines in the placental plasma of placental malaria-positive women including: tumor necrosis factor alpha (TNF-*α*), interferon gamma (IFN-*γ*), and interleukin-2 (IL-2). Accordingly, several studies have reported presence of T cells in the IVS of women with placental malaria [28, 29]. Apart from cytokines and chemokines secreted by maternal monocytes and T cells, studies in chronic-infected placentas have implicated the role of B cells in placental inflammatory response during placental malaria [29, 30]. Therefore, sequestration of IE in the IVS and subsequent inflammation induces pathological changes that may limit placental capacity to transfer nutrients to the growing fetus, therefore increasing risk of having LBW babies. The histopathological changes induced during placental malaria include ST degradation and destruction of the villous integrity [31], decrease in villous surface areas [32], fibrinoid necrosis [26, 30], and thickening of trophoblast basement membrane [26, 30].

### 2.2. Response of Trophoblasts to Malaria

In placenta infected with malaria, ST are exposed to IE, malaria pigment (hemozoin), maternal immune cells, and inflammatory cytokines. Studies on response of ST to malaria have mostly been done* in vitro*. Using primary trophoblasts and the BeWo cell line, Lucchi et al. [33, 34] showed that binding of CSA-positive Plasmodium parasites to BeWo stimulated BeWo to secrete cytokines including macrophage inhibitory factor (MIF), macrophage inhibitory protein- (MIP-) 1*α*/CCL3 (detected at the protein level), and transforming growth factor- (TGF-) *β* (at the transcriptional level). In addition, Lucchi et al. also demonstrated that hemozoin could stimulate ST to secrete chemokines, CXCL8, CCL3, CCL4, and a cytokine, TNF-*α* as well as soluble intracellular adhesion molecule-1 (ICAM-1) [33]. Furthermore, data from the same group showed that binding of IE to ST induced phosphorylation of trophoblasts proteins [35]. These studies suggest that ST respond to IE and natural hemozoin by secreting cytokines and chemokines commonly found in IVS of placenta malaria-positive women. Moreover, the phosphorylation of trophoblast proteins following IE binding implies that changes in the regulation of protein functions are induced. However, IE may produce other soluble and insoluble bioactive molecules to which ST might respond. More studies identifying the response of ST to IE bioactive molecules are needed.

## 3. Placental Malaria and Syncytiotrophoblast Functions

### 3.1. Immunological Protection

ST forms a physical barrier, separating maternal and fetal blood. Under normal circumstances, ST also prevents hematogenous transmission of infection from mother to the fetus including placental-*P. falciparum-*IE. The barrier has additional antimicrobial activities: synthesizing high levels of nitric oxide synthase, producing microvillus-associated glycosaminoglycans, and secreting antiviral interferons [36]. The fetal trophoblasts create an immune-active barrier to pathogens. Like innate immune cells, trophoblasts express pattern recognition receptors (PRR), including Toll-like receptors (TLR1-10) [37–39] and nucleotide-binding oligomerization domain (NOD) receptors including inflammasomes [40]. While TLRs recognize and respond to both extracellular and endosomal pathogen-associated molecular patterns (PAMPs), NOD receptors are cytosolic sensors and, therefore, recognize intracellular PAMPs. Studies on human trophoblasts have shown that TLR expression on trophoblasts differs with gestation age, with certain types of TLRs being differentially expressed in the first, second, or third trimesters (reviewed in [41]). This temporal expression of TLRs across gestation suggests that different responses are produced by trophoblasts to various TLR ligands at different points in gestation. For example, TLR6 is only expressed by third trimester trophoblasts and since TLR2 forms heterodimers with either TLR1 or 6 upon stimulation, TLR6 ligands may not induce a response earlier in gestation. Furthermore, while TLR2/TLR1 stimulation leads to apoptotic death in the first trimester trophoblast, the ligation of the same ligand through TLR2/TLR6 heterodimers prevents cell death in human third trimester primary trophoblasts [42]. As* P. falciparum* has a number of well-characterized PAMPs such as glycosylphosphatidylinositol- (GPI-) anchor, which can activate TLR2/TLR1 and TLR2/TLR6 heterodimers (reviewed in [43]), the temporal expression of TLRs may explain the differential placental pathology observed during placental malaria throughout gestation. This hypothesis is supported by population based study in pregnant women which demonstrated that having malaria infection in the first trimester increased the risk of miscarriage compared to having malaria infection in late gestation [44].

Other PAMPs associated with malaria include malarial hemozoin and parasite DNA. Studies by Coban et al. [16] established that the malarial hemozoin is a TLR9 ligand and it has since been proposed to activate the NLRP3 inflammasome [17]. Generally, PRR-expression by the fetal trophoblasts aids in combating pathogens in the IVS; however the resulting immune responses they induce may contribute to poor neonatal outcomes associated with sequestered parasites during placental malaria. Although TLRs are essential innate receptors at the maternal fetal interface, the immediate response of ST to* P. falciparum*-derived PAMPs cannot be studied* in vivo*. However, the role of TLRs in some pathological pregnancies has been reported [45]. Therefore the question still remains: Does ST response to* Plasmodium* derived PAMPs via PRRs induce pathophysiological changes that lead to FGR? In other words do* P. falciparum*-associated PAMPs influence ST function?

### 3.2. Transport and Metabolism

Syncytiotrophoblast, the major transporting epithelium in the placenta, is polarized with the ability for uptake and transfer of nutrients, such as amino acids, glucose, fatty acids, minerals, cholesterol, and some xenobiotics. The apical surface of ST is perfused by maternal blood, whereas the basal membrane is in intimate contact with fetal capillary networks. The ST regulates maternal-fetal transport of nutrients, including glucose, amino acids, lipids, gases, and ions. Owing to its syncytial nature, transplacental transfer of nutrients, for example, amino acids and glucose, is made possible by carrier proteins that are expressed on both apical and basal membranes of the ST. Generally, the driving force of the transport of most nutrients through ST depends on concentration and an electrochemical gradient (e.g., glucose and ions). However, another form of transport operates against the concentration gradient of solutes and thus requires energy in terms of hydrolysis of adenosine-5′-triphosphate (ATP). Amino acids transporters require energy in the form of ATP to transfer amino acids from the apical surface to the basal membrane of ST [37].

Fetal growth is dependent upon the ST transporting activities of various transporters. Accordingly, fetuses with restricted growth often have low plasma concentrations of amino acids and are hypoglycemic [46]. This association has been reported by Jansson et al. [47], who have shown that the activities of several transporters are dysregulated in pregnancies associated with idiopathic FGR, including transporters for system A amino acids (SLC38A), leucine, sodium-dependent and -independent taurine, Na^+^/K^+^-ATPase, Ca^2+^, and Na^+^/H^+^ exchanger. Recently, it was described that the SLC38A2 and SLC38A1 transcripts were downregulated in women with placental malaria with inflammation [48] suggesting that the uptake of amino acids by fetal cells is impaired during placental malaria. It should be noted that nutrients taken up by the fetal ST influence metabolism and subsequent nutrient delivery to the fetus. The transfer of nutrients across ST is influenced by the uteroplacental and umbilical blood flow and regulated by various fetal, maternal, and placental signals [49]. Several placental proteins, placental signaling molecules, and placental hormones synthesized and secreted by the placenta facilitate transport of nutrients across ST (reviewed in [49]), influencing fetus growth.

### 3.3. Regulation of Vasculogenesis and Angiogenesis

Vasculogenesis and angiogenesis are processes essential for creating and maintaining uteroplacental blood flow and, therefore, influence the exchange of nutrients between mother and fetus. While angiogenesis refers to the formation of new blood vessels from preexisting ones, vasculogenesis is the formation of blood vessels from endothelia cells. The main factors produced by ST that regulate vasculogenesis and angiogenesis by fetal endothelia cells are vascular endothelial growth factors (VEGF), placental growth factor (PIGF), and transforming growth factor (TGF) beta [50]. VEGF signals via two specific receptors, VEGFR-1 and VEGFR-2, by inducing the production of nitric oxide (NO) from trophoblasts. The literature suggests that VEGF, PIGF, and their respective receptors are important for regulating trophoblast survival and angiogenesis [51]. VEGF induces angiogenesis and regulates vasculogenesis and, therefore, placental vasculature. Other molecules that perform angiogenic functions during pregnancy are placental angiopoietins-1 (ANGPT-1 and -2). The ANGPTs are expressed by trophoblasts and induce their effect via receptor kinases,* Tunica* internal endothelial cell kinase-2 (Tie-2) receptor expressed on fetal endothelial cells. ANGTP-Tie forms a vascular-specific ligand/receptor system that controls endothelia cell survival and vascular maturation of placental and is therefore important for fetal growth (reviewed in [52]).

Dysregulation of angiogenesis has been implicated in placental-malaria-associated FGR from* in vivo* studies by Dorman et al. [53, 54] and others [55]. VEGF transcription levels are reported to be lower in ST from placental malaria-positive-placentas as compared to ST from asymptomatic placentas [56]. In addition, Neres et al. [57] implicated vasculogenesis impairment in pregnant* P. berghei*-ANKA-positive mice. Furthermore, studies in the same mouse model showed dysregulation of angiopoietins by placental malaria and this correlated with FGR. These animal studies are supported by cross-sectional and longitudinal studies in pregnant women from endemic areas where the ANGPT-2 was elevated and ANGPT-1 decreased in placental malaria-positive women and correlated with having LBW babies [55, 58]. Other studies have shown that levels of antiangiogenic molecules, which are known to be produced by ST, are elevated during placental malaria, including the fms-like tyrosine kinase-1 (sFLT1), a soluble vascular endothelia growth factor receptor that sequesters VEGF-1 [59] and soluble endoglin [60]. Previously, genome wide expression studies using the BeWo cell line [61] demonstrated that placental malaria-associated cytokines dysregulated biological processes associated with placental vasculogenesis and angiogenesis suggesting that inflammatory response in the IVS during placental malaria interferes with blood flow, a functional aspect of ST. Therefore, alterations in the production of angiogenic factors by ST during placental malaria could lead to poor vascularisation of the placenta and hence impair fetal growth.

### 3.4. The Insulin-Like Growth Factor Signaling (IGF-1) Axis: Regulation of Transplacental Transfer of Nutrients, Cells Survival, and Proliferation

The insulin-like growth factor (IGF) axis consists of two polypeptide hormones, IGF-1 and IGF 2, cell surface receptors IGF-1R and IGF-2R, soluble IGF-binding proteins, and an IGF-binding protease, which all control growth of many organs, including the placenta. During pregnancy, the IGF are mainly secreted by the placental cells including ST and the growing fetus. IGF-1 is an important growth pathway for regulation of placental and fetus growth throughout gestation; it plays a major role in the transfer of nutrients across the placenta through ST, facilitating cell survival and proliferation [62, 63]. Gene-disruption studies show that mice carrying the IGF-1 mutant allele have a 40% reduced birth weight. In human, mutation in the IGF-1 gene reduces fetus growth by 60% and subsequent postnatal growth [64]. On the other hand, IGF-R2 gene targeting in mice resulted in placental and fetus overgrowth [65]. In addition, Laviola et al. [66] found a decrease in the expression of IGF-1R and signaling molecules in human intrauterine FGR restricted-placentas. These observations indicate that impairment of both IGF-I action through the absence of IGF expression and IGF signaling molecules may lead to abnormal fetus and placental growth.

Dysregulation of the IGF axis has been studied in LBW babies and pathological pregnancies due to various causes. Chiesa et al. [67] investigated the relationship between reduced maternal IGF-1 and FGR and found that IGF-1 concentration was lower in pregnancies with FGR than in normal birth weight babies [67]. In addition, preeclamptic patients exhibit alterations of IGF binding protein-1 and IGF-2 at the fetus: maternal interface [68]. More recently, it has been shown that IGF-1 concentrations in the cord blood of neonates born to placental malaria-positive mothers are lower than those born to mothers without placental malaria and these positively correlated with birth weight [68]. In the referred study, maternal IGF-1 concentration was positively correlated with placental malaria with inflammation. Furthermore,* in vitro* studies by Kidima [61] using a placental cell line (BeWo cells) showed that malaria-associated cytokines and chemokines but not intact placental-binding-*P. falciparum* IE dysregulated IGF1 pathway supporting the observation by Umbers and group [68]. Dysregulation of the IGF1 pathway during placental malaria may interfere with the transfer of nutrients across the placenta and therefore affect fetal growth.

### 3.5. The Mammalian Target for Rapamycin (mTOR) Pathway: Regulator of Placental and Fetus Growth

#### 3.5.1. The mTOR

mTOR is a large serine threonine kinase (originally named as a target of rapamycin, a bacteria toxin that inactivated the kinase in yeast) which belongs to the phosphoinositide 3-kinase related kinase (PI3K) and is constitutively expressed in eukaryotic cells which function to control growth (reviewed in [69]) [70, 71]. mTOR exists in two functional complexes, mTOR complex 1 (mTORC1) which is sensitive to rapamycin and mTOR complex 2 (mTORC2) which is not [72]. mTOR functions to facilitate and promote cellular growth and metabolism in response to diverse extracellular and intracellular signals [72, 73]. For instance, the activities of mTORC1 are regulated by energy status, hormones, growth factors, amino acids, glucose, and oxygen [72, 74], while those of mTORC2 can be activated by the growth factors and amino acids.

The mTOR signaling pathway regulates critical for cellular growth processes including protein and lipid synthesis, regulation of cellular metabolism and ATP production (reviewed in [74]), angiogenesis (reviewed in [75]), and cytoskeleton organization [69]. In addition mTOR controls cell survival by inhibiting apoptosis, facilitating cell proliferation and actin organization and negatively regulating autophagy. Consequently, mTOR is important in normal physiological functioning of several tissues and cells [71]. It follows, therefore, that dysregulation of the mTOR pathway has been reported to play a major role in several pathological events including cancer, metabolic diseases such as obesity and type two diabetes, and neurodegenerative diseases (reviewed in [74]).

#### 3.5.2. Placental mTOR and Fetus Growth

Placenta mTOR is the regulator of placental and fetus growth. Placental mTOR protein, which is expressed in ST [76], is critical in regulating fetal growth by influencing transplacental transport of nutrients, predominantly amino acids. Evidences are showing that growth factors, hormones, nutrients, such as amino acids, glucose [76–78] energy, and stress [78] regulate the placental mTOR activities just like in other cells/tissues. Studies using human trophoblastic cells by Roos et al. [79] reported that activation of amino acid transporting activities by the growth factors, IGF-1, and insulin, occurred through the mTOR signaling pathway. Furthermore, evidence shows that mTOR stimulate expression and amino acid transporters in human placenta, including system A and L, as well as taurine transporters [79, 80], and therefore enhance nutrient delivery to the fetus. Consequently, mTOR signaling activities were reported to be reduced in FGR placentas [81], but the mTOR protein expression increased, suggesting occurrence of adaptive response.

*Does Poor Maternal Nutrition Stir Placental Malaria Pathogenesis: Is the Placental mTOR the Target?* The role of mTOR in the pathogenesis of malaria is starting to unravel. For instance, it has been shown that inhibition of mTOR signaling by treating* Plasmodium berghei* ANKA-infected mice with mTOR inhibitor rapamycin prevented pathology in an experimental cerebral malaria [82]. Specifically, the rapamycin* Plasmodium berghei*-positive mice had lower parasite density, less accumulation of IE in the brain, and therefore no brain dysfunction. However, the mTOR inhibitor failed to impede inflammation. Further research on the role of mTOR is necessary in placental malaria animal model, such as the baboon, where* Plasmodium knowlesi* have been shown to sequester to the placenta [83]. On the other hand,* in vitro* studies using the BeWo cell line exposed to malaria-associated cytokines plus chemokines (secreted by human monocytic cell line (THP1) cells that were incubated with* P. falciparum* infected red blood cells) induced dysregulation of mTOR pathway [61], suggesting that mTOR pathway in ST might be interfered by placental malaria-inflammatory-cytokines. Furthermore, using placental malaria-infected placentas, Dimasuay et al. [84] showed a dysregulation of mTOR in the placenta with chronic inflammation further supporting an observation that placental malaria with inflammation may deregulate the mTOR pathway.

However, the placental mTOR links maternal nutrient availability and fetus growth [76]. Also the availability of nutrients to the fetus entirely depends on the functional-placental growth pathways and hormones which signal to the mTOR signaling pathway. On the other hand, the expression of mTOR is influenced by nutrition such as amino acids [85]. Interestingly, many epidemiological studies in malaria endemic areas have implicated the association between maternal nutrition and poor-neonatal outcome in women with placental malaria. For example, cohort studies by Landis et al. [86] reported a 2- to 8-fold increase in risk of FGR in women with placental malaria that were undernourished compared to those who were well nourished. Furthermore, longitudinal studies by Griffin et al. [87] showed an increase in uterine artery resistance amongst the undernourished women who had early-pregnancy-*P. falciparum*-parasitemia compared to those who had early parasitemia but were well nourished, suggesting placental insufficiency. The increase in uterine resistance among placental malaria-positive women who were undernourished suggests that malaria may hamper the adaptive responses which have been reported to occur in preeclamptic women or that had idiopathic FGR [88]. So the question remains: is the pathogenesis of placental malaria that leads to FGR stirred by lack of nutrients in the IVS of placenta? And is placental mTOR the target?

**Figure 2:**
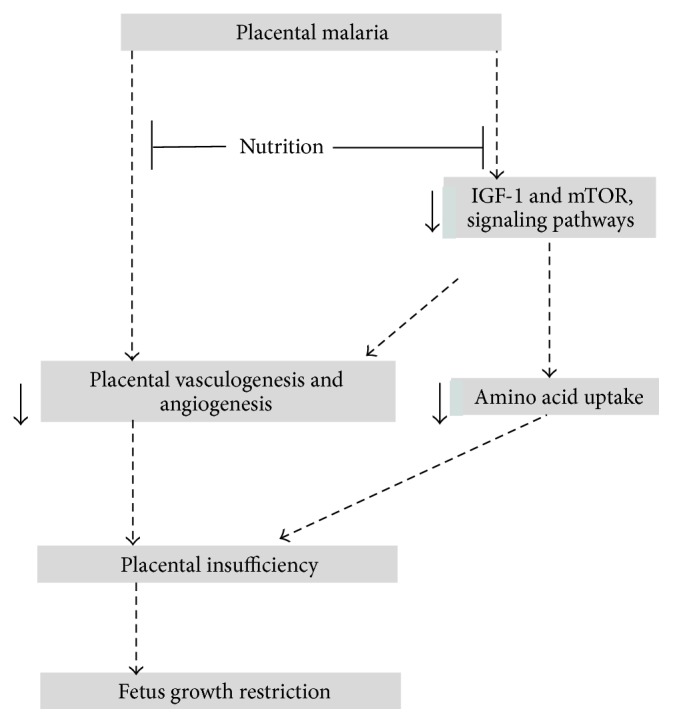
Model explaining biological mechanisms associated with the development of fetal growth restriction as a result of placental malaria. Both* in vivo* and* in vitro* studies have documented dysregulation of vasculogenesis and angiogenesis in placental malaria. Moreover, the IGF-1 and mTOR growth pathways that link maternal nutrients to fetal nutrient availability are downregulated. In addition, nutrient transporters, particularly system A amino acid transporters, are downregulated during placental malaria and therefore limit amino acid uptake. Interestingly, epidemiological studies indicate nourishment or food availability during pregnancy significantly override the effect of placental malaria on neonatal outcome (FGR). We hypothesize that supplementing pregnant women with allowable level of amino acids may override the effect of malaria on neonatal outcome (↓ downregulate).

It follows therefore that since (i) nutrients activate the mTOR pathway [89] and (ii) that mTOR links important growth pathways, IGF-1, VEGF, and insulin pathways, to regulate fetus growth* in utero* and (iii) that activation of amino acid transporting activities by the growth factors, IGF-1 and insulin, occurred through the mTOR signaling pathway [79], we hypothesize that lack of nutrients in the IVS during pregnancy might stir up pathogenesis of placental malaria and hence FGR by downregulating the mTOR signaling pathway (Figure 2). It is therefore recommended that studies on potential interventional approach for placental malaria during pregnancy should consider intermittently supplementing placental malaria-high-risk-women with allowable levels of essential amino acids and other micronutrients in malaria endemic areas.

## 4. Conclusion

Placenta malaria dysregulates aspects of ST functions, which associate with delivering maternal nutrients to the fetus* in utero,* therefore increasing the risk of FGR. Establishing the role of placenta malaria on ST function aids in understanding the pathophysiology induced by placental malaria and has implications in finding focus that could lead to the prevention of poor neonatal outcomes. Nutrition may override the effects of placental malaria on ST functions and therefore improve neonatal outcome during placental malaria. The role of maternal nutrition in placental malaria pathogenesis has an implication in future malaria interventional approaches.
